# The impact of diversity management on innovative work behavior: the mediating role of employee engagement in an emerging economy

**DOI:** 10.3389/fsoc.2024.1441109

**Published:** 2024-09-04

**Authors:** Abdallah M. Elamin, Hazem Aldabbas, Ahmed Zain Elabdin Ahmed

**Affiliations:** Department of Management, University of Science and Technology of Fujairah, Fujairah, United Arab Emirates

**Keywords:** diversity management, innovative work behavior, employee engagement, social exchange theory, UAE, innovation, diversity

## Abstract

This study explored how diversity management fuels innovative employee behavior in the United Arab Emirates’ (UAE) emerging economy. Surveying 120 individuals from various service organizations, the research found a strong connection between diversity initiatives, and employee engagement, ultimately leading to more innovative practices. Diversity management directly impacts employee engagement, which in turn significantly influence innovative work behaviors. Interestingly, employee engagement fully mediates the relationship between diversity and innovation. These findings highlight the crucial role of diversity management in fostering a culture of innovation. Organizations can leverage this by investing in training by equipping employees with skills to effectively engage across diverse teams as well as promoting inclusivity through cultivating a work environment that values and respects differences, fostering open communication and collaboration. By implementing these recommendations, organizations can harness the power of diverse perspectives to drive innovation and gain a competitive edge.

## Introduction

In the contemporary business landscape, every organization aims to sustain its operations, cultivate a highly productive workforce, and generate profits (Moltz, 2021). The key to achieving a successful and efficient business lies in the establishment of effective policies and procedures by management to organize and oversee employee activities and behavior. The behavior of employees plays a crucial role in forecasting success, emphasizing the importance of starting performance enhancement initiatives internally (Indeed Editorial Team, 2021). Well-trained employees exhibit a range of desirable qualities such as competence, responsiveness, and reliability (Kotler et al., 2017). A positive work environment characterized by supportive behaviors significantly impacts organizational performance, leading to increased morale and heightened productivity, engagement, and innovation among employees. Conversely, negative work environments have detrimental effects on organizational performance, manifesting in high turnover rates, absenteeism, and various other adverse outcomes (All Things Talent Team, 2020).

The concept of diversity has evolved to include intentional goals to improve organizational performance and effectiveness (Joubert, 2017). Diversity management (DM) is based on a new perspective of differences as strategic assets that can be managed to create a competitive edge (Vincent et al., 2024). Hence, it is viewed as a resource by some organizations as it is regarded as a springboard for innovation and best business practices, while others see it as a weakness that negatively influences performance.

Employee innovation refers to creating, promoting, and implementing innovative and practical ideas to improve the performance of individuals, organizations, or businesses (West and Farr, 1990). Staff innovation can contain components of responsibility that are part of the prescribed tasks and new work outside of formal position descriptions (West, 2002; Potocnik and Anderson, 2016). Furthermore, to increase employee creativity and innovation, firms should consider crucial characteristics such as a diverse workforce (Luu, 2019), and employee engagement (Waheed et al., 2017; Hapsari et al., 2019), One of the most effective ways to create ultimate rivalry is to recognize current employees, who are becoming increasingly varied and diverse. That is why massive efforts in supporting rules and procedures are critical to ensure the inclusion of employees from diverse backgrounds and move those employees forward to achieve some beneficial outcomes such as increased profits, employee engagement, and organizational growth (Downey et al., 2015).

The majority of past research on diversity management and innovative work behavior (IWB) focused on the higher education industry (e.g., Ganji and Johnson, 2020), service industry (e.g., Downey et al., 2015; Bizri, 2018; Hapsari et al., 2019), and government (e.g., Bizri, 2018; Hapsari et al., 2019). Given the scarcity of research, it is interesting to investigate how diversity management is linked to innovative work behavior, and employee engagement, in the UAE’s emerging economy. As a result, this study is being conducted across a sample of private and governmental institutions in the UAE to investigate the impact of diversity management on innovative work behavior. The study also investigates how employee engagement influence the relationship between diversity management and innovative work behavior.

### UAE background

The United Arab Emirates is a perfect example of a vibrant emerging economy. While its initial success was due to large oil reserves (The World Bank, 2023), the UAE realized the limitations of a resource-based economy and embarked on a strategic diversification plan (International Monetary Fund, 2022). This included investments in non-oil sectors such as tourism, finance, and logistics, resulting in a more resilient and knowledge-based economy (The World Bank, 2023). This transition was accelerated by the UAE’s strategic location and infrastructure development, which established it as a worldwide commerce hub (Murphy, 2009). The UAE government actively fosters research and development, with the goal of positioning the country as a hub for future technologies. However, obstacles persist, such as reliance on oil and hiring competent labor. Despite this, the UAE’s dedication to diversification, innovation, and human capital development prepares it for long-term success in the global economy (World Economic Forum, 2023). The UAE’s population of more than 9.992 million comprises people from more than 200 nationalities, which have contributed to the tremendous development of the economy and enriched it with diverse knowledge. Moreover, the availability of people from various backgrounds can be considered a valuable source of information to produce powerful insights creating new innovative ideas. Since it was formed, the UAE has internationally reinforced the root of peace, security, and growth (The United Arab Emirates' Government Portal, 2021). One of the strengths that makes it unique is its high tolerance and respect for others derived from following the teachings of Islam and cultural habits.

The paper is structured into six sections. In section 2, the literature and conceptual framework are examined, introducing key concepts, definitions, and the connections between model variables. Hypotheses are formulated based on previous research findings, and a hypothetical research model is proposed based on the literature review discussions. Section 3 covers the methodology and data collection process, including details about the approach, participants, measurements and procedures. Subsequently, Section 4 presents the findings. Section 5 discusses the outcomes of the study. Finally, Section 6 provides a summary of the study’s conclusion, limitations, and further research.

## Literature review

### Theoretical background

Diversity refers to variances in people’s identities that might have an impact on their lives, both as customers and employees (Robbins and Judge, 2013). Diversity management is the practice of managing disparities among people to maximize effectiveness and efficiency in the workplace (Edwin, 2002; Vincent et al., 2024). It is referred to as designing and implementing practical activities that aim to improve the expected benefits of diverse persons in the workplace (Groeneveld and Van de Walle, 2010). The actions related to diversity management are part of the organization’s human resource department, which aims to foster greater workplace inclusion (Personio, 2022). Workplace diversity has a significant impact on management practice. Managers should detect and respond to differences in ways that promote employee retention and productivity.

The concept of employee engagement is defined as an employee’s involvement with, satisfaction with, and excitement for performing job tasks that he or she does (Robbins and Judge, 2013). Abraham (2012) defined worker engagement as the extent to which an employee is emotionally connected to the company’s achievements. Employee engagement can also be defined as having favorable feelings that lead to aptitude, affective, and social repercussions for an organization (Kahn, 1990; Gatenby et al., 2009), or the majority of effort available for usage displayed by employees in their work (Frank et al., 2004). Furthermore, highly engaged individuals are passionate about their employment and have a strong bond with the people in their organizations. Robbins and Judge (2013) succinctly states that engagement becomes a real issue for most organizations.

Innovation is the transformation of an idea into a practical solution that adds value from the client’s perspective (Skillicorn, 2016). According to Drucker (1985), innovation rarely results from a burst of motivation in business. It stems from a cold-eyed examination of seven categories of opportunities. He also saw innovation as a specific function of entrepreneurship, whether in public sector institutions or enterprises in general. Janssen (2000) defines innovative work behavior as the deliberate production, introduction, and use of innovative ideas inside a work role, group, or company with the goal of improving role performance, the group, or the company. Furthermore, it refers to an individual’s intentional act of creating and executing innovative ideas and commodities, known as technical innovation, or new practices and procedures, known as organizational innovation (Van de Ven, 1986; West and Farr, 1989).

### Hypotheses development

#### Relationship between diversity management and innovative work behavior

IWBs play a crucial role in achieving competitive advantages and ensuring organizational success (Aldabbas et al., 2021). According to Van der Vegt and Janssen (2003) and Bassett-Jones (2005), efficient management of workforce diversity can lead to increased innovation and creativity in businesses. According to Gupta (2011), workplace diversity is associated with increased creativity, innovation, and competitive advantage. Similarly, Syed et al. (2021) contend that DM improves IWB through employee involvement and affective commitment. Meanwhile, Hapsari et al. (2019) reported that DM results in positive effect on IWB based on a field study conducted in Indonesia. Chen et al. (2019) discovered a positive relationship between cognitive diversity and IWB. Additionally, other studies, such as Lambert (2016) and Shin et al. (2017), showed that the diverse workforce has been crucial in boosting the creative behavior of personnel and working teams. In view of the preceding theoretical reasons in previous literature, this study constructs its first hypothesis as follows:

*H*1: Diversity management is positively related to innovative work behavior.

#### Relationship between diversity management and employee engagement

A few previous studies have associated the issue of diversity management with employee engagement, such as Downey et al. (2015), who, by implementing a questionnaire offered to 4,597 employees working in the health services industry, verified that having evident diversity management initiatives and strategies plays a substantial role in increasing employees’ engagement at work (Alshaabani et al., 2022). Downey et al. (2015) empirical study demonstrated the positive impact of diversity management programs on work engagement. Prior research has also shown that implementing diversity guidance was a highly effective strategy for increasing employee engagement among Australian people (Skalsky and McCarthy, 2009). Therefore, it is possible to address the following hypothesis:

*H*2: Diversity management is positively related to employee engagement.

#### Relationship between employee engagement and innovative work behavior

A prior study found that when individuals are highly engaged, they exhibit more creativity (Aldabbas et al., 2023) and innovative work behavior in the workplace (Agarwal et al., 2012; Prieto and Pérez-Santana, 2014). Assuming an employee is content with his or her employment, he or she is more likely to appreciate thinking outside the box, discussing experiments, developing creative job skills, or engaging in innovative activities (Agarwal et al., 2012). An empirical study for 327 employees working in the aviation industry in the UAE found that work engagement relates positively and significantly to IWB (Saeed AlShamsi et al., 2023). Similarly, an empirical study of 372 Chinese senior employees across various industries finds that employee engagement is positively related to IWB (Ali et al., 2022). Consequently, engagement predicts innovative activity. Based on the prior debates, the following hypothesis is proposed:

*H*3: Employee Engagement is positively related to Innovative Work Behavior.

#### Mediation effect of employee engagement between diversity management and innovative work behavior

As previously stated, various research has found that diversity management is an important predictor of employee engagement (Skalsky and McCarthy, 2009; Downey et al., 2015). Furthermore, research has demonstrated that employee engagement can influence and predict innovative work behavior (Agarwal et al., 2012; Prieto and Pérez-Santana, 2014). Based on these recommendations, appropriate diversity management initiates a social exchange connection that reinforces employee engagement, which, in turn, leads to and promotes innovative work behavior on the employee side. As a result, it is reasonable to predict that when employees believe their firm is following adequate diversity management programs and procedures, they will become more involved and reciprocate by demonstrating innovative work behavior. Therefore, we hypothesize the following in the UAE context:

*H*4: Employee engagement mediates the relationship between diversity management and innovative work behavior.

### Hypothetical research model

**Figure 1:**
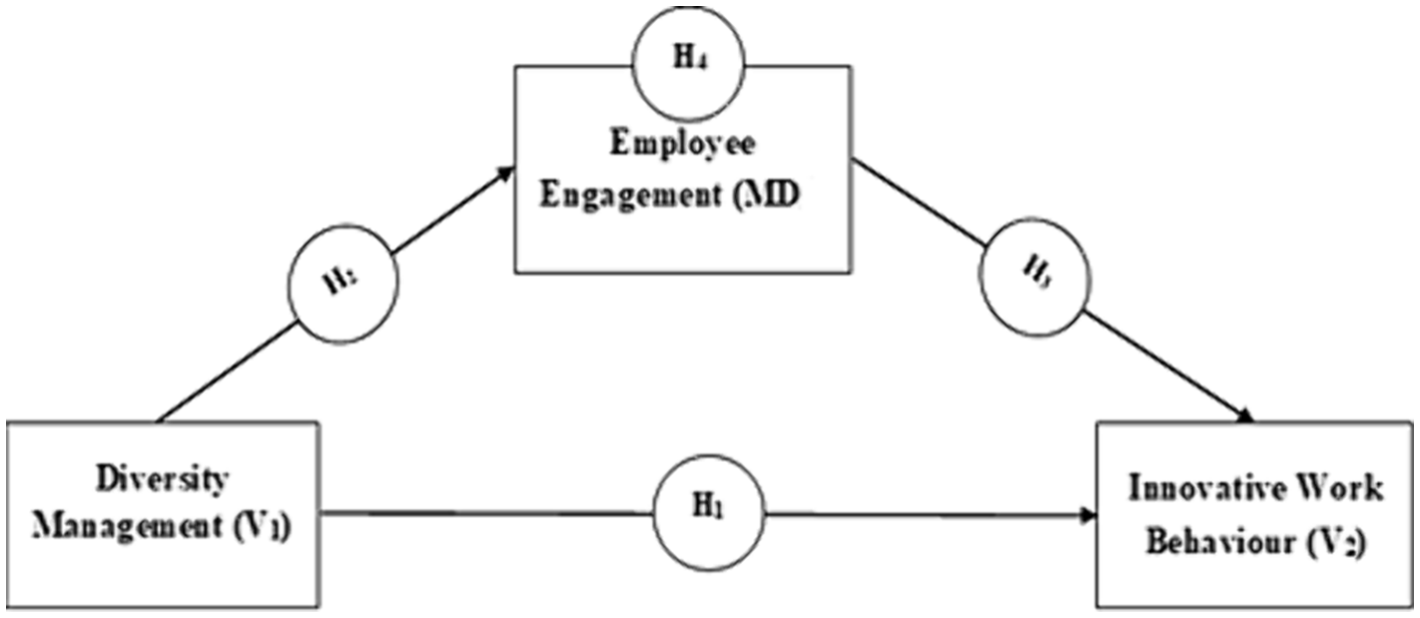
Hypothetical research model.

Following the hypotheses development, I suggest the following hypothetical research model (Figure 1).

## Research methodology

### Approach

The research design of this study is centered on surveys and utilizes cross-sectional data. Cross-sectional data has become a prevalent method in recent research for evaluating causal relationships (Han et al., 2018; Ohunakin et al., 2019). The study adopted a positivist approach to investigate the connections between diversity management, innovative work behavior, and the specified mediating variable.

### Participants

This study, conducted in a cross-sectional manner, aimed to explore the causal relationships between the variables under investigation among employed individuals. Data collection in the Middle East region is often deemed challenging and is associated with various methodological constraints (Elamin and Tlaiss, 2015). As a result, a convenience sample was utilized for this research. The questionnaire was disseminated among individuals employed in the service sector of the UAE. The service industry was selected due to its diverse range of economic activities, including commercial services, financial institutions, higher education, and government agencies. The survey was administered using an online platform, with distribution commencing in January 2024 and concluding in February 2024. Participation in the survey was voluntary, and all participants provided written, informed consent to partake in the study. The response rate stood at 64.0%, with 200 surveys distributed and 128 responses received. However, due to inconsistencies in response patterns and incomplete surveys, only 120 surveys were deemed suitable for inclusion in this study.

### Measurements

The questionnaire comprised 31 questions sourced from related research utilized in this survey, categorized into four groups: demographic information, diversity management, employee engagement, and innovative work behavior. To assess diversity management, a six-item scale proposed by Bizri (2018) was employed, using a seven-point Likert scale ranging from zero (strongly disagree) to six (strongly agree). The Utrecht Work Engagement Scale, a shortened version with nine items developed by Schaufeli and colleagues, was used to measure employee engagement. This scale includes three sub-scales: vigor, dedication, and absorption, each with three items rated on a 7-point Likert scale from 0 (never) to 6 (always). In measuring innovative work behavior, Kanter (1988) stages of innovation, as utilized by Janssen (2000), were employed. Kanter’s measures consisted of three items each for idea generation, promotion, and realization, combined into a single variable for innovative work behavior. This variable was also assessed on a seven-point scale ranging from 0 (never) to 6 (always), by aggregating the three items. Cronbach’s alpha values indicated a high level of consistency and reliability among responses for each participant across all constructs. The reliability coefficients (α) for diversity management (α = 0.908), employee engagement (α = 0.951), and innovative work behavior (α = 0.938) were substantial, as shown in Table 1.

**Table 1:** Means, standard deviation, and intercorrelations among study variables.

Variables	M	SD	1	2	3
1. Diversity management	5.08	0.92	(0.908)		
2. Employee engagement	5.13	1.03	0.764**	(0.951)	
3. Innovative work behavior	5.01	0.95	0.571**	0.808**	(0.938)

Source: Field Survey (2024). *p < 0.05; **p < 0.01; ***p < 0.001.

### Procedures

Considering that Arabic serves as the official language of the UAE, administering the questionnaire solely in its original English form would have significantly impacted the response rate. Therefore, back-translation techniques were employed to develop an equivalent Arabic version. Following the guidance of Tlaiss (2013), two translators independently translated the English questionnaire into Arabic, and then two other independent translators back-translated the Arabic version into English. The initial and back-translated versions were compared by language experts and the authors, proficient in both languages, resulting in minor adjustments mainly focused on grammatical appropriateness. The final Arabic version was piloted with a group of students, and no further modifications were made after this phase. As part of the procedures employed in this study, both descriptive statistics and hierarchical regression analysis were utilized to describe the study variables and test the hypotheses. The statistical analysis was conducted using SPSS version 29.

## Findings

### Sample description

The data collected was analyzed using SPSS version 29.0. Table 2 displays the distribution of the study sample based on demographic information, which includes gender, nationality, age, organizational tenure, job rank, and level of education. In Table 2, there were 88 males (73.3%) and 32 females (26.7%). The sample consisted of 30.8% Emirati participants and 69.2% non-Emirati participants. The majority of research participants fell into the age group of 21 to 29 years old (82.5%), while the remaining participants were aged 30 years and older. Additionally, Table 2 indicated that 70% of participants had less than 3 years of organizational tenure, with most being employees at the first level. In terms of education level, 93.3% of participants held a bachelor’s degree or lower, while only 6.7% possessed a Master or Ph.D. degree.

**Table 2:** Research sample demographic profile.

	Frequency	
Variables	n (total – 120)	% (total – 100%)
Gender		
Male	88	73.3
Female	32	26.7
Nationality		
Emirati	37	30.8
Non-Emirati	83	69.2
Age		
21–29	99	82.5
30–34	6	5.0
35–39	6	5.0
40 and above	9	7.5
Organizational tenure		
Less than 3 years	84	70.0
3 - less than 6 years	26	21.7
6 - less than 10 years	0	0
10 years and more	10	8.3
Job rank		
Employee	87	72.5
Assit. Mgr./supervisor/Chief Employee	8	6.7
General manager	4	3.3
Senior manager	1	0.8
Director	2	1.7
Other	18	15.0
Level of education		
High school	11	9.2
Diploma	4	3.3
Bachelor’s degree	97	80.8
Graduate (Masters/Ph.D.)	8	6.7

Source: Field Survey (2024).

### Descriptive results

In order to fulfill the study’s objective of examining diversity management, employee engagement, and innovative work behavior among UAE employees, descriptive analysis was carried out. Table 1 provides a summary of the mean, standard deviation, Pearson intercorrelations, and reliability scores for the study variables. Regarding diversity management, the findings revealed a high overall level of employee perception in this area, with a mean score of 5.08. Similarly, the level of employee engagement was found to be high, as indicated by a mean score of 5.13. Additionally, the results regarding innovative work behavior showed that employees had a positive response to this variable, with a mean score of 5.01.

The results provide initial support for the majority of hypotheses, specifically H1, H2, and H3. The correlations observed were statistically significant at the 0.01 level among the variables examined in the study. Initial hypothesis testing utilized the Pearson correlation statistical test. As depicted in Table 1, the analysis demonstrates a positive correlation between diversity management and employee engagement (*r* = 0.764, *p* < 0.01), as well as between diversity management and innovative work behavior (*r* = 0.571, *p* < 0.01). These findings lend support to the first and second hypotheses. Additionally, there was a strong positive association observed between employee engagement and innovative work behavior (*r* = 0.808, *p* < 0.01), further substantiating the H3 hypothesis.

### Hypotheses testing

To investigate the relationship between diversity management and innovative work behavior (H1), hierarchical regression analysis was conducted in two stages. The first step involved entering control variables related to demographics, such as age, organizational tenure, job rank, and level of education, into the analysis. Subsequently, in the second step, the diversity management variable was introduced. The outcomes of this analysis are presented in Table 3. The hierarchical regression analysis revealed that diversity management effectively predicted innovative work behavior. As depicted in Table 3, diversity management accounted for an average of 34.5% of the variance in innovative work behavior. It was found that diversity management significantly influenced innovative work behavior (*β* = 0.611, *p* < 0.001). Furthermore, upon introducing diversity management in step 2, there was a notable 0.334 change in R^2^ [*F*(5,114) = 13.550, *p* < 0.001]. These results fully support H1, which examines the linear relationship between diversity management and innovative work behavior.

**Table 3:** Results of hierarchical regression testing the relationship between diversity management and innovative work behavior (DM—IWB).

Criterion variables		
	Innovative work behavior	
Predictors	ΔR^2^	β
Step 1: Controls	0.039	
Age		−0.248
Organizational tenure		0.078
Job rank		−0.028
Level of education		0.102
Step 2:	0.334***	
Diversity management		0.611***
N	120	
Adjusted *R*^2^	0.345	
Equation *F*-value	13.550***	

Source: Field Survey (2024). *β*, Standardized beta. ***p < 0.001.

To examine the correlation between diversity management and employee engagement (H2), hierarchical regression analysis was carried out in two stages. Initially, control variables related to demographics, such as age, organizational tenure, job rank, and level of education, were included in the analysis. Subsequently, in the second step, the diversity management variable was introduced. The outcomes of this analysis are summarized in Table 4. The hierarchical regression analysis revealed that diversity management effectively predicted employee engagement. As demonstrated in Table 4, diversity management accounted for an average of 56.9% of the variance in employee engagement. It was found that diversity management significantly influenced employee engagement (*β* = 0.769, *p* < 0.001). Furthermore, upon introducing diversity management in the second step, there was a substantial 0.528 change in R^2^ [*F*(5,114) = 32.436, *p* < 0.001]. These results fully support H2, which explores the linear relationship between diversity management and employee engagement.

**Table 4:** Results of hierarchical regression testing the relationship between diversity management and employee engagement (DM—EE).

Criterion variables		
	Employee engagement	
Predictors	ΔR^2^	β
Step 1: Controls	0.070	
Age		−0.059
Organizational tenure		−0.119
Job rank		−0.014
Level of education		0.217
Step 2:	0.528***	
Diversity management		0.769***
N	120	
Adjusted *R*^2^	0.569	
Equation *F*-value	32.436***	

Source: Field Survey (2024). *β*, standardized beta. ***p < 0.001.

To explore the relationship between employee engagement and innovative work behavior (H3), hierarchical regression analysis was conducted in two stages. Initially, control variables related to demographics, including age, organizational tenure, job rank, and level of education, were included in the analysis. Subsequently, in the second step, the employee engagement variable was introduced. The findings of this analysis are summarized in Table 5. The hierarchical regression analysis revealed that employee engagement significantly contributed to innovative work behavior. As indicated in Table 5, employee engagement accounted for an average of 67.1% of the variance in innovative work behavior. It was observed that employee engagement had a substantial impact on innovative work behavior (*β* = 0.829, *p* < 0.001). Furthermore, upon introducing employee engagement in the second step, there was a notable 0.646 change in R^2^ [*F*(5,114) = 49.475, *p* < 0.001]. These results strongly support H3, which examines the linear relationship between employee engagement and innovative work behavior.

**Table 5:** Results of hierarchical regression testing the relationship between employee engagement and innovative work behavior (EE-IWB).

Criterion variables		
	Innovative work behavior	
Predictors	Δ*R*^2^	*β*
Step 1: controls	0.039	
Age		−0.248
Organizational tenure		0.078
Job rank		−0.028
Level of education		0.102
Step 2:	0.646***	
Employee engagement		0.829***
N	120	
Adjusted *R*^2^	0.671	
Equation *F*-value	49.475***	

Source: Field Survey (2024). *β*, standardized beta. ***p < 0.001.

Finally, the mediating effect of employee engagement on the association between diversity management and innovative work behavior (H4) was examined using Baron and Kenny (1986) four-step approach for establishing mediation. The outcomes are outlined in Table 6. In the first step, it was revealed that diversity management significantly and positively predicted innovative work behavior (*β* = 0.571, *p* < 0.001). Moving to the second step, diversity management and employee engagement showed a positive and robust relationship (*β* = 0.764, *p* < 0.001). Upon entering employee engagement into the regression model in the third step, it was observed to have a significant impact on innovative work behavior (*β* = 0.891, *p* < 0.001). However, the β coefficient for the relationship between diversity management and innovative work behavior became negative and insignificant, experiencing a substantial decline (*β* = −0.109, *p* = 0.195). Ultimately, the results of the fourth step confirmed that employee engagement fully mediated the connection between diversity management and innovative work behavior.

**Table 6:** Results of the hierarchical regression testing the mediating effect of employee engagement in the relationship between diversity management and innovative work behavior.

Criterion variable		
	Employee engagement	Innovative work behavior
Predictor	Β	β
Step 1		
Diversity management	0.764***	0.571***
Adjusted *R*^2^	0.580***	
Step 2		
Diversity management		−0.109
Employee engagement		0.891***
Adjusted *R*^2^		0.652***
Δ*R*^2^		0.658
F for Δ*R*^2^ (Steps 1 and 2)		112.461***

Source: Field Survey (2024). *β*, standardized beta. ***p < 0.001.

## Discussion

The purpose of this study was to investigate the relationship between diversity management and innovative work behavior, as well as the mediating effect of employee engagement in the relationship between DM and IWB in the context of the UAE’s emerging economy. The study collected data through a survey and employed statistical techniques to examine its hypotheses. The findings supported all the hypotheses proposed in the current study.

The first hypothesis, which demonstrated the effect of diversity management on innovative workplace behavior, was confirmed. Correlations between diversity management perspective and innovative work behavior were shown to be both positive and significant. The findings of hierarchical regression supported the first hypothesis at a significant level. These findings are consistent with those of Chrobot-Mason and Aramovich (2013), Lambert (2016), Shin et al. (2017), Ganji et al. (2021), and Syed et al. (2021). Diversity management promotes innovative work behavior by promoting inclusion and making equal opportunities available throughout the organization. Additionally, diversity management helps promote respectful behavior among personnel and allows them to support each other regardless of their background. Furthermore, Diversity encourages greater exploring for unique information and perspectives. Thus, it leads to developing the ability to make smart decisions and solve problems at work.

In terms of the relationship between diversity management and employee engagement, as indicated in hypothesis two, this study found a strong and substantial association between these two variables. These findings are consistent with prior research investigating the impact of diversity management on employee engagement (Skalsky and McCarthy, 2009; Downey et al., 2015; Alshaabani et al., 2022). Downey et al. (2015) found that diversity management strategies had a favorable impact on employee engagement in the workplace. He discovered that instituting diversity practices by service firms offers each employee the feeling that the organization cares about them and values their cultural differences (Downey et al., 2015). As a result, every organization will experience better levels of employees’ engagement. In the sense that when an organization acknowledges and implements a diversity management program for its employees, it is more likely to receive increased participation and engagement (Kahn, 1990). Such a line of reasoning can be interpreted in terms of Blau (1964) Social Exchange Theory (SET), which is regarded as a viable explanatory framework for employee-organization interaction. Social exchange theory offers a reasonable explanation for employee-organization interdependence. According to SET, if employees believe their employer supports them by implementing diversity management programs, they are more likely to reciprocate by engaging in more work that benefits the organization.

The findings of the current study also validated the third hypothesis, which stated that employee engagement has a positive and significant influence on innovative work behavior. This finding was consistent with those of Agarwal et al. (2012), Prieto and Pérez-Santana (2014), and Saeed AlShamsi et al. (2023). For instance, Agarwal et al. (2012) and Prieto and Pérez-Santana (2014) argued that enhancing employee engagement in the workplace results in innovative working behavior. They are exposed to researching and utilizing problem-solving, particularly while working in service companies, and as a result, they are more likely to achieve creative ideas, promote and use them, or display innovative work behaviors (Zhang and Bartol, 2010; (Agarwal et al., 2012). Thus, highly engaged employees are more inclined to seek out ways to innovate, whether it results in a better customer experience, more profitability, or improved quality.

Though our findings contribute to the existing literature suggesting that diversity management has a direct impact on innovative work behavior, this effect might not be unconditional. Our findings on the mediating effects of employee engagement indicate that, as hypothesis four states, employee engagement has a strong full mediating influence on the relationship between diversity management and innovative work behavior. According to SET and reciprocity norms (Gouldner, 1960), employees that receive a high level of commitment from their organization for diversity management initiatives reciprocate by increasing their engagement in the organization, which can lead to more innovative work behavior. Prior research has confirmed that diversity management leads to employee engagement (Skalsky and McCarthy, 2009; Downey et al., 2015; Alshaabani et al., 2022), as well as being a predictor of innovative work behavior (Agarwal et al., 2012; Prieto and Pérez-Santana, 2014; Kim and Ko, 2017).

## Conclusion, limitations, and further research

Although extensive research has been conducted on diversity management, employee engagement, and innovative work behavior in Western countries, there is a lack of research in the Africa and Middle East (AME) context, including the UAE. This study offers empirical evidence for the correlation between diversity management, employee engagement, and innovative work behavior in the UAE’s emerging economy. Our findings suggest that employee engagement can serve as a bridge between diversity management and innovative work behaviors. These results have significant implications both theoretically and managerially.

Theoretically, this study contributes to the existing literature on diversity management, employee engagement, and innovative work behavior in several ways. Firstly, it provides empirical support for the validity of three crucial constructs: diversity management, employee engagement, and innovative work behavior. It also examines the applicability of these constructs beyond North American settings, as the research was conducted using a sample from the UAE. Secondly, there has been limited exploration of the relationship between diversity management, employee engagement, and innovative work behavior in non-Western contexts, especially in the AME region. This research adds substantial value to the existing knowledge by demonstrating that employees’ perceptions of diversity management influence their engagement, which in turn promotes innovative behavior, particularly within the UAE context. This highlights the importance of diversity initiatives, employee engagement, and support in fostering innovative work behavior within organizations. Thirdly, the connection between diversity management and innovative work behavior has not received enough attention in non-Western contexts. This study reveals that employee engagement plays a crucial role as an intermediary in this relationship. Effective diversity management programs and practices enhance employee engagement, leading to increased participation and ultimately fostering innovative work behavior.

Additionally, it is crucial to consider the practical managerial implications of these findings for managers and how these insights can contribute to enhanced organizational effectiveness. Firstly, managers in UAE organizations should acknowledge that employees’ perceptions of diversity management practices significantly influence their level of engagement and their inclination to demonstrate innovative behavior. Consequently, managers must adjust their decisions and actions accordingly to align with these insights. Secondly, to promote innovation, organizations should strengthen their investments in diversity management programs and strategies for employee engagement. Managers in UAE organizations can adopt innovative approaches to enhance diversity management, such as broadening their talent pool through unconventional recruitment methods, creating bias-free job descriptions, fostering an inclusive culture, and providing comprehensive diversity training. Nart et al. (2018) have established a direct correlation between diversity management and leaders’ competence and perceptions. Leaders proficient in implementing diversity management practices can cultivate a culture of dedication, engagement, and heightened creativity within their organizations. Similarly, managers must prioritize promoting employee engagement by fostering a culture of recognition and appreciation, creating a supportive and positive work environment based on trust and effective communication, and encouraging collaboration and teamwork among employees.

While this study offers valuable insights into the correlation between diversity management and innovative work behavior, as well as the mediating role of employee engagement, it is essential to note several limitations that can guide future empirical research. Firstly, the study’s small sample size (only 120 respondents) limits the generalizability of the findings. Future studies should aim for broader generalization by incorporating randomly sampled data from the entire employee population in the UAE. Secondly, the convenience sampling method employed in obtaining the sample introduces potential selection bias. The author attempted to mitigate this bias by controlling demographic and organizational variables. Thirdly, the study relied on cross-sectional data, which hinders the ability to establish causality among variables. Therefore, future research should consider employing longitudinal analysis to address this limitation. Additionally, the study solely relied on self-reported data collection, which may lead to common method variance and potentially impact result accuracy (Podsakoff et al., 2003). To minimize this, researchers should consider employing multiple data collection approaches.

## Data availability statement

The raw data supporting the conclusions of this article will be made available by the authors, without undue reservation.

## Ethics statement

Ethical review and approval was not required for the study on human participants in accordance with the local legislation and institutional requirements. The studies were conducted in accordance with the local legislation and institutional requirements. Written informed consent for participation was not required from the participants or the participants' legal guardians/next of kin in accordance with the national legislation and institutional requirements. The questionnaire included an introductory statement explaining the study's purpose, and participants' completion of the questionnaire was considered as implied consent. No identifying information was collected, ensuring participants' privacy and confidentiality.

## Author contributions

AE: Conceptualization, Data curation, Formal analysis, Investigation, Methodology, Project administration, Visualization, Writing – original draft. HA: Investigation, Methodology, Writing – review & editing. AA: Supervision, Writing – review & editing.
